# Supplementary material for: Evolutionary Analysis of the Land Plant-Specific TCP Interactor Containing EAR Motif Protein (TIE) Family of Transcriptional Corepressors
Source: Plants (Basel). 2025 Aug 5;14(15):2423. doi: 10.3390/plants14152423 (PMC12349606; doi:10.3390/plants14152423)
Supplement: Supplementary file 1 [file plants-14-02423-s001.zip › plants-3709965-supplementary.pdf]

**Figure S1: Density plot of Arabidopsis *TCP* and *TIE* gene expression.**

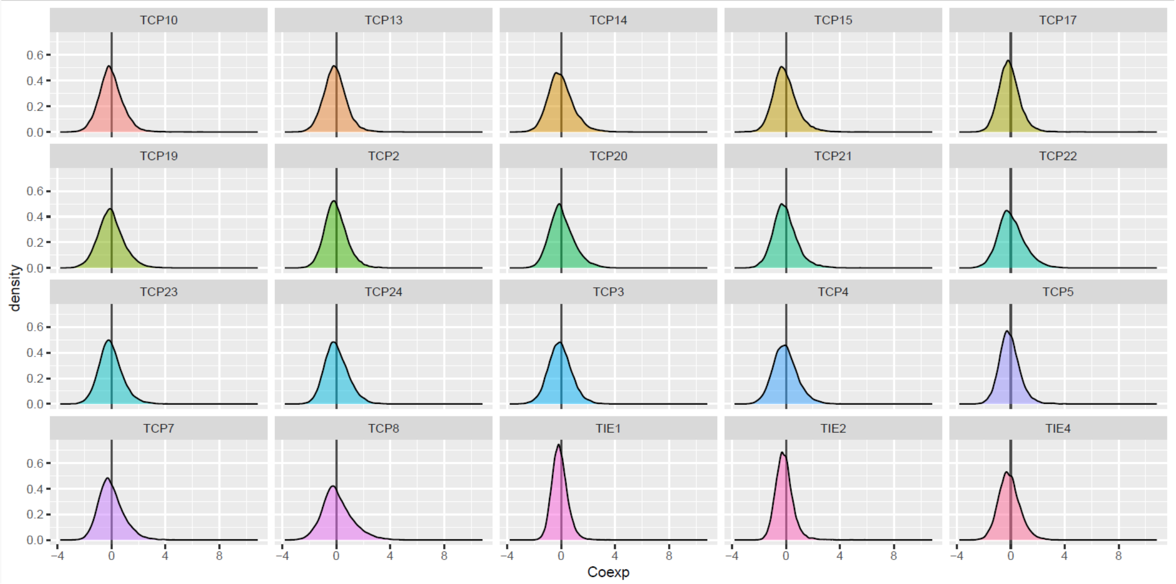

**Figure S2: Density plot of rice *TCP* and *TIE* gene expression.**

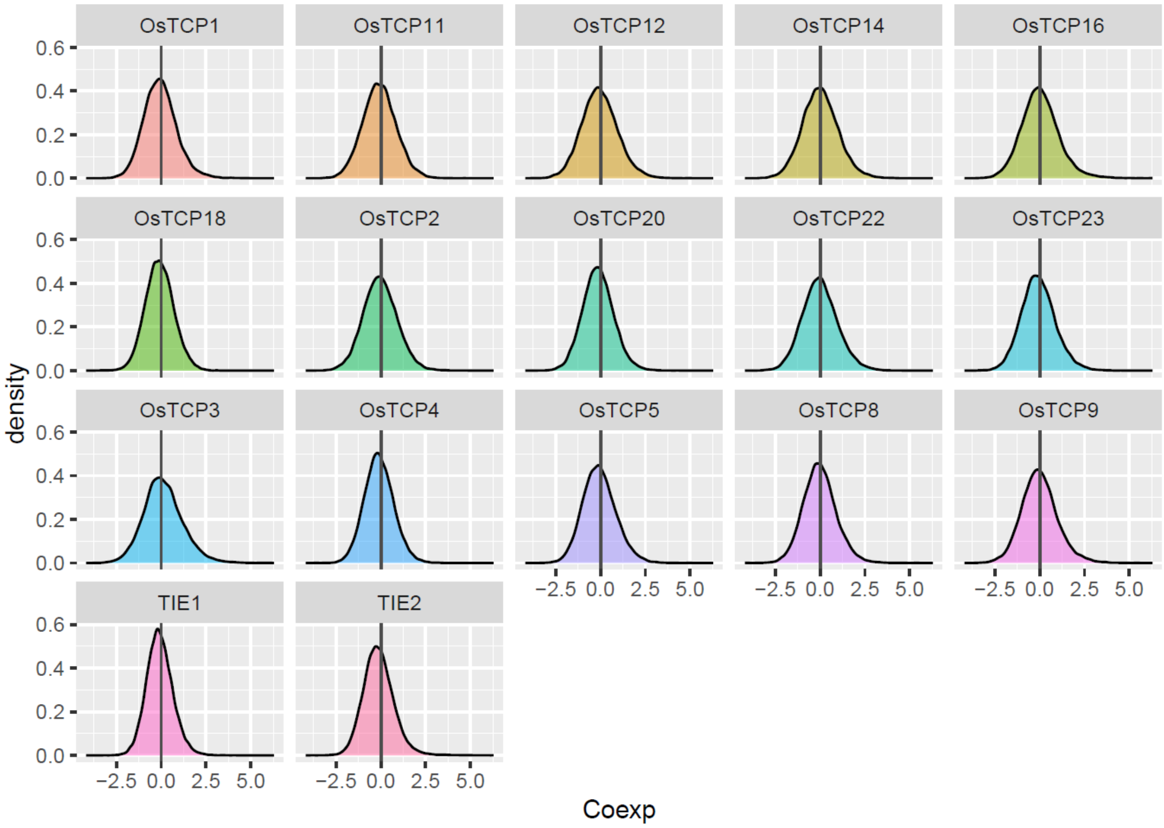

**Table S1. List of species and sequences included in the phylogenetic analyses.**

| | **Species** | **Sequences** |
|---|---|---|
| Basal embryophyte | *Ceratodon purpureus* | CepurR40.9G047000.1 |
| | | CepurR40.4G027600.1 |
| | *Marchantia polymorpha* | Mapoly0019s0053.1 |
| | *Selaginella moellendorffii* | S.moellendorffii_438336 |
| | | S.moellendorffii_411605 |
| | | S.moellendorffii_410500 |
| | *Sphagnum fallax* | Sphfalx06G039900.1 |
| | | Sphfalx09G033400.1 |
| | *Physcomitrium patens* | Pp3c1_13420V3.1 |
| | | Pp3c2_26200V3.1 |
| | | Pp3c4_31500V3.1 |
| Basal angiosperm | *Amborella trichopoda* | AmTrH1.05G012400.1 |
| | *Cinnamomum kanehirae* | CKAN_01161600 |
| | | CKAN_00636200 |
| | *Liriodendron tulipifera* | Litul.15G088600.1 |
| | | Litul.01G167900.1 |
| Eu-dicot | *Anacardium occidentale* | Anaoc.0013s0631.1 |
| | | Anaoc.0793s0009.1 |
| | | Anaoc.0591s0001.1 |
| | | Anaoc.0019s0481.1 |
| | *Aquilegia coerulea* | Aqcoe7G164000.1 |
| | *Arabidopsis lyrata* | AL3G48010.t1 |
| | | AL1G42700.t1 |
| | | AL4G29890.t1 |
| | *Arabidopsis thaliana* | AT4G28840.1 |
| | | AT2G20080.1 |
| | | AT1G29010.1 |
| | | AT2G34010.1 |
| | *Brassica oleraceae* | Bol033553 |
| | | Bol045903 |
| | | Bol020836 |
| | *Cammelina sativa* | Csa11g014710.1 |
| | | Csa12g020570.1 |
| | | Csa10g013740.1 |
| | | Csa15g085670.1 |
| | | Csa19g048770.1 |
| | | Csa17g040730.1 |
| | | Csa03g032100.1 |
| | | Csa07g007340.1 |
| | | Csa05g021290.1 |
| | *Capsella rubela* | Carub.0007s1180.1 |
| | | Carub.0003s3436.1 |
| | | Carub.0001s2719.1 |
| | | Carub.0004s1565.1 |
| | *Carica papaya* | evm.model.supercontig_23.57 |
| | | evm.model.supercontig_131.73 |
| | *Crambe hispanica* | Crahi.0215s0060.1 |
| | | Crahi.0195s0029.1 |
| | | Crahi.0236s0017.1 |
| | | Crahi.2176s0001.1 |

| | | |
|---|---|---|
| | | Crahi.0188s0030.1 |
| | *Glycine max* | Glyma.01G145600.1 |
| | | Glyma.05G229100.1 |
| | | Glyma.08G036100.1 |
| | | Glyma.09G193400.1 |
| | *Gossypium hirsutum* | Gohir.A05G025200.1 |
| | | Gohir.D05G026700.3 |
| | | Gohir.A06G064800.1 |
| | | Gohir.D06G063400.2 |
| | *Junglans regia* | Jr01_28090_p1 |
| | | Jr02_22940_p1 |
| | | Jr09_13690_p1 |
| | | Jr02_21310_p1 |
| | *Linum usitatissimum* | Lus10012982 |
| | | Lus10034960 |
| | | Lus10013688 |
| | *Manihot esculenta* | Manes.03G024200.1 |
| | | Manes.16G112500.1 |
| | *Phaseolus vulgaris* | Phvul.002G311400.1 |
| | | Phvul.004G168350.1 |
| | *Populus trichocarpa* | Potri.004G052900.1 |
| | | Potri.018G084100.1 |
| | | Potri.006G161100.1 |
| | | Potri.001G375600.1 |
| | *Silene alba* | Sialb.0079s0293.1 |
| | | Sialb.0008s0753.1 |
| | | Sialb.1741s0004.1 |
| | | Sialb.0013s0432.1 |
| | | Sialb.0687s0015.1 |
| | | Sialb.0002s0496.1 |
| | | Sialb.0039s0465.1 |
| | | Sialb.2917s0007.1 |
| | | Sialb.1502s0023.1 |
| | | Sialb.0004s1474.1 |
| | *Solanum tuberosum* | Soltu.DM.12G025110.1 |
| | | Soltu.DM.07G007480.1 |
| | *Theobroma cacao* | Thecc.09G105500.1 |
| | | Thecc.06G176400.1 |
| | | Thecc.07G103600.2 |
| Monocot | *Anana comosus* | Aco000375.1 |
| | | Aco004428.1 |
| | | Aco026961.1 |
| | *Brachypodium distachyon* | Bradi2g06832.3 |
| | | Bradi2g46460.1 |
| | | Bradi2g16858.1 |
| | *Joinvillea ascendens* | Joasc.06G109000.1 |
| | *Mussa acuminata* | GSMUA_Achr2T15330_001 |
| | *Oryza sativa* | LOC_Os01g11430.1 |
| | | LOC_Os01g48530.1 |
| | | LOC_Os05g48570.1 |
| | *Setaria italica* | Seita.5G148600.1 |
| | | Seita.5G269100.2 |
